# EFFECTS OF PSYCHOEDUCATION ON BURDEN, DEPRESSION, AND ANXIETY IN DEMENTIA FAMILY CAREGIVERS: A SYSTEMATIC REVIEW

**DOI:** 10.1093/geroni/igae098.4170

**Published:** 2024-12-31

**Authors:** Hyeyeon Shin, Chanchanok Wandee, Donal O’Mathuna

**Affiliations:** Ohio State University, Columbus, Ohio, United States; Ohio State University, Ohio State University, Ohio, United States; Ohio State University, Ohio State University, Ohio, United States

## Abstract

As dementia cases rise globally, caregiving becomes increasingly prolonged and demanding compared to other chronic illnesses, leading to heightened risks of caregiver burden, depression, and anxiety. Evaluating psychoeducation is crucial as it provides caregivers with knowledge, coping strategies, and support to manage their mental health and caregiving responsibilities. This review assesses the effectiveness of psychoeducation interventions on caregiver burden, depression, and anxiety among family caregivers of dementia patients. Following PRISMA guidelines, we searched PubMed, CINAHL, MEDLINE, and PsycINFO using relevant terms and assessed study quality with the Joanna Briggs Institute (JBI) tool. We included only randomized controlled trials (RCTs) where the control group received usual care or other interventions. We reviewed 23 articles involving 2,651 informal caregivers. All articles evaluated caregiver burden, with 11 studies showing statistically significant reductions. Depression was assessed in 16 articles, with 8 demonstrating statistically significant positive effects, while anxiety was measured in 9 articles, with 4 reporting significant improvements. Among these, one study targeting a specific population showed significant effects on both burden and depression. In another study, although overall depression scores in the intervention group were not statistically significant, a subgroup with scores above the cutoff line showed positive effects, whereas the control group’s outcomes worsened. Overall, 50% of the studies reported significant positive effects of psychoeducation. Despite some studies lacking statistical significance, reductions in scores indicate that psychoeducation can alleviate caregiver burden, depression, and anxiety. Further research and development of targeted interventions for vulnerable populations are needed.

